# Exploring obesity, physical activity, and digital game addiction levels among adolescents: A study on machine learning-based prediction of digital game addiction

**DOI:** 10.3389/fpsyg.2023.1097145

**Published:** 2023-03-03

**Authors:** Mehmet Gülü, Fatma Hilal Yagin, Ishak Gocer, Hakan Yapici, Erdem Ayyildiz, Filipe Manuel Clemente, Luca Paolo Ardigò, Ali Khosravi Zadeh, Pablo Prieto-González, Hadi Nobari

**Affiliations:** ^1^Department of Coaching Education, Faculty of Sport Sciences, Kirikkale University, Kirikkale, Türkiye; ^2^Department of Biostatistics and Medical Informatics, Inonu University Faculty of Medicine, Malatya, Türkiye; ^3^Graduate School of Health Sciences, Ankara University, Ankara, Türkiye; ^4^Sports Management Department, Faculty of Sport Sciences, Tekirdağ Namık Kemal University, Tekirdağ, Türkiye; ^5^Escola Superior Desporto e Lazer, Instituto Politécnico de Viana do Castelo, Rua Escola Industrial e Comercial de Nun’Álvares, Viana do Castelo, Portugal; ^6^Instituto de Telecomunicações, Delegação da Covilhã, Lisboa, Portugal; ^7^Department of Teacher Education, NLA University College, Oslo, Norway; ^8^Department of Exercise Physiology, Faculty of Sport Sciences, University of Isfahan, Isfahan, Iran; ^9^Health and Physical Education Department, Prince Sultan University, Riyadh, Saudi Arabia; ^10^Faculty of Sport Sciences, University of Extremadura, Cáceres, Spain; ^11^Department of Motor Performance, Faculty of Physical Education and Mountain Sports, Transilvania University of Braşov, Braşov, Romania

**Keywords:** sedentary behaviors, obesity, body mass index, addiction, children, physical inactivity

## Abstract

Primary study aim was defining prevalence of obesity, physical activity levels, digital game addiction level in adolescents, to investigate gender differences, relationships between outcomes. Second aim was predicting game addiction based on anthropometric measurements, physical activity levels. Cross-sectional study design was implemented. Participants aged 9–14 living in Kirikkale were part of the study. The sample of the study consists of 405 adolescents, 231 girls (57%) and 174 boys (43%). Self-reported data were collected by questionnaire method from a random sample of 405 adolescent participants. To determine the physical activity levels of children, the Physical Activity Questionnaire for Older Children (PAQ-C). Digital Game addiction was evaluated with the digital game addiction (DGA) scale. Additionally, body mass index (BMI) status was calculated by measuring the height and body mass of the participants. Data analysis were performed using Python 3.9 software and SPSS 28.0 (IBM Corp., Armonk, NY, United States) package program. According to our findings, it was determined that digital game addiction has a negative relationship with physical activity level. It was determined that physical activity level had a negative relationship with BMI. In addition, increased physical activity level was found to reduce obesity and DGA. Game addiction levels of girl participants were significantly higher than boy participants, and game addiction was higher in those with obesity. With the prediction model obtained, it was determined that age, being girls, BMI and total physical activity (TPA) scores were predictors of game addiction. The results revealed that the increase in age and BMI increased the risk of DGA, and we found that women had a 2.59 times greater risk of DGA compared to men. More importantly, the findings of this study showed that physical activity was an important factor reducing DGA 1.51-fold. Our prediction model Logit (P) = 1/(1 + exp(−(−3.384 + Age*0.124 + Gender-boys*(−0.953) + BMI*0.145 + TPA*(−0.410)))). Regular physical activity should be encouraged, digital gaming hours can be limited to maintain ideal weight. Furthermore, adolescents should be encouraged to engage in physical activity to reduce digital game addiction level. As a contribution to the field, the findings of this study presented important results that may help in the prevention of adolescent game addiction.

## Introduction

In the last decade, digital games have become one of the most spent online activities among adolescents, and simultaneously, this activity has changed from being partly computer or console-based to a multiplatform activity (Association, 2015). With the proliferation of smart mobile phones, mobile games are one of the most popular entertainment sectors of the multimedia application industry in Europe (Consumer, 2015). With the increase in games that can be played with technological devices such as computers, phones, and tablets, research on the positive and negative aspects of games has also increased. Research shows that active video games and some games can be a motivating source for following an active lifestyle and can help improve indicators of health status in young adults (Zurita-Ortega et al., 2018). However, in addition to the positive effects of the developing digital technology, it causes many problems on health in terms of mental, physical, social, environmental aspects that may cause problems such as not being able to limit its use or using it for some unintended activities. Spending excessive time on digital games in the ongoing daily routine leads to adopting a sedentary lifestyle and creating an antisocial structure (Christakis, 2019; Almourad et al., 2020).

Adolescence is a unique development period of human development and the foundations of a healthy life are laid. According to the definition of the World Health Organization, adolescence is the life stage between childhood and adulthood, between the ages of 10 and 19. Adolescents experience rapid physical, cognitive and psychosocial growth. This influences what adolescents think, how they feel, and how they make decisions, and most importantly, how they interact with the world around them (WHO, 2022). The level of physical activity is insufficient in adolescents and children all over the world, especially female adolescents and decreases with age (Aubert et al., 2021).

Promoting a lifelong healthy lifestyle and participation in physical activity is important for many countries (Pühse and Gerber, 2005). In order to prevent these problems and improve quality of life, physical activity is recommended by WHO. For children, physical activities of moderate to vigorous intensity (such as jumping rope, line games, holding and rolling games, ice skating, gymnastics, skiing, athletics, soccer, swimming, dancing, table tennis, and slow-paced cycling) should be preferred for at least 60 min every day, at least three times a week. In its current guidelines, the WHO recommends physical activity for children and adolescents, including moderate-to-high intensity, mostly aerobic exercise, for at least 60 min a day on average throughout the week. High-intensity aerobic exercise should also be practiced at least 3 days a week to improve bone and muscle health (WHO, 2010, 2019). Physical activity, which is defined as all body movements that increase the activity of the circulatory system and cause fatigue, performed in a way that causes energy expenditure with our skeletal muscles above the basal level (Bronikowska et al., 2021). A physically active lifestyle begins to develop in childhood and continues throughout life with moderate or high stability of physical activity (Telama et al., 2014). Studies on the health benefits of physical activity have reported that physical activity has positive results in terms of academic achievement and musculoskeletal health (Strong et al., 2005). However, inadequacy of physical activity not only causes many problems in adolescents, but also negatively affects their physical development (Storz, 2020). However, studies have reported that physical activity level tends to decrease during adolescence (Dumith et al., 2011). To further promote physical activity, the World Health Organization launched in 2018 the project of more active people for a healthier world, new global action on physical activity, to reduce the global prevalence of insufficient physical activity among adolescents and adults by 15% by 2030 (WHO, 2019). The rate of physical activity declines significantly during adolescence, and girls are less physically active than boys (56% vs. 39%; Kumar et al., 2015). This coincides with the increase in obesity rates among 11–15-year-old adolescents, of whom 38% are overweight (van Jaarsveld and Gulliford, 2015).

Increases in overweight and obesity have become an important public health problem worldwide (Wyatt et al., 2006; Abarca-Gómez et al., 2017). Since the 1970s, there has been an almost three-fold increase in the prevalence of obesity worldwide, and studies have reported that approximately one-fifth of American children are obese (Ogden et al., 2018). Although its positive effects are known, it is stated that approximately 80% of children and adolescents aged 13–18 worldwide do not participate in physical activity (Rhodes et al., 2017). Studies have reported an increase in poor eating habits in adolescents (Abarca-Gómez et al., 2017) and a decrease in physical activity, especially during the pandemic period (Kohl et al., 2012). Problems such as the habit of being sedentary, attitudes and behaviors of not doing physical activity, and nutritional disorders that start in childhood and adolescence continue as the age progresses, causing individuals not to include physical activity adequately in their lives (Pombo et al., 2020). Both inactive lifestyle and poor eating habits are thought to be among the leading causes of overweight and obesity prevalence (Stevens et al., 2012). In a similar study, it was found that obese children had higher levels of food addiction and lower physical activity levels than non-obese children (Gülü et al., 2022). Although efforts to prevent obesity have increased in the last two decades worldwide, these efforts have not been enough to prevent the increase in obesity in children (Brown et al., 2019). Traditional interventions aimed at preventing obesity, targeting energy balance and individual health behavior change, such as calorie restriction and increasing the level of physical activity, were limited in reducing the prevalence of obesity (Al-Khudairy et al., 2017; Mead et al., 2017).

The study reported that children aged 12–18 years who spent a lot of time with computers and television had higher BMI values (Alghadir et al., 2021). Increased body composition has a significant association with physical activity (especially increased daily moderate to vigorous physical activity), with just over 30% of children aged 8–12 years playing digital games for 2 h or more daily (O’Brien et al., 2021). Li et al. (2014) found that among 1,150 rural and urban middle school students with digital addiction in China, obesity values (32.92%) differed from those of students without digital addiction (21.06%). Therefore, they found a relationship between obesity and internet addiction and reported that internet addiction is an independent risk factor for obesity (Li et al., 2014). According to a study, 14 of 26 studies (53%) published between 2013 and 2018 reported that there was no relationship between video games and obesity, while 12 reported a positive relationship (Kracht et al., 2020). When the studies in the literature are examined, the relationship between obesity and game addiction remains unclear.

To our knowledge, this is the first study to predict gaming addiction using machine learning based on physical activity and BMI. Our hypothesis that as the level of game addiction increases, the prevalence of obesity will increase due to the decrease in physical activity level and increase in BMI. The primary aim of this study was to define the prevalence of obesity, physical activity levels and digital game addiction level in adolescents, and to investigate gender differences and relationships between outcomes. The second aim was to predict game addiction based on anthropometric measurements and physical activity levels. The concept of “digital addiction,” which emerged with the impact of technological developments, has become a major and widespread problem today. Due to the temporary solutions and the ineffectiveness of some preventive approaches, it is predicted that the concept of physical activity will be more effective as an indispensable prevention method in the lives of individuals.

## Materials and methods

### Study design and setting

This cross-sectional study was implemented in line with the purpose described in the STROBE checklist (von Elm et al., 2007). A simple random sampling method was used in the data collection process. All the procedures described below were conducted in October/November 2021.

### Participants

With G*Power software (University of Dusseldorf, Dusseldorf, Germany, version 3.0.1), the independent sample *t*-test was used to calculate sample size and actual power (*α* = 0.05, power = 0.80, effect size = 0.35). The results revealed that with a sample size of 260 participants, the actual power was 80.2% (Faul et al., 2007). The research group of this study consisted of (231 girls %57 and 174 boys %43) 405 adolescent individuals in Turkey. The participants mean age, height, and body mass were 11.37 ± 1.45 years, 149.42 ± 11.17 cm, and 44.22 ± 13.06 kg, respectively. The research was announced in schools through a poster or verbal and information was given to the participants who wanted to take part in the research. Inclusion criteria: it consists of individuals between the ages of 9 and 14, speaking fluent Turkish, currently residing in Kirikkale and without any mental or chronic disease that prevents them from participating in physical activity. After reading the information form about the research by the families and children, the participants filled out the questionnaires after measuring the height and body mass. Participants who wanted to withdraw from the study could withdraw from the study at any time without completing the questionnaire. This study was approved by the Kirikkale University Social and Human Sciences Ethics Committee in line with the Declaration of Helsinki (Protocol no: 10/18.10.2021).

### Data collection method

A descriptive survey model and a quantitative method were used in the research (Karasar, 2016). Because of the Cronbach Alpha reliability analysis of the study, it was concluded that the game addiction for adolescents and physical activity inventories for children was reliable in the range of 0.70–0.90. PAQ-C Cronbach’s Alpha 0.781, Game addiction scale Cronbach’s Alpha 0.902 was detected. The data collection method consisted of three parts: The first part was the personal information form, which consisted of questions about gender, age, health status. Second part was evaluation anthropometric measurements were made at the school sport center. All anthropometric measurements were taken in the afternoon and the indoor at sport center ambient temperature was recorded as temperature 22°. In the third part PAQ-C and Digital Game addiction scales were applied. Height was measured using a sensitive measuring up to 0.1 cm (Seca 217, Seca, Hamburg, Germany). During height measurement, the participant was standing, without shoes, with heels and his head in the horizontal plane. Body mass was measured using the sensitive Tanita body analysis system (Tanita Corp., Tokyo, Japan) up to 0.1 kg for a participant. To determine the physical activity status of the participants, Erdim et al. (2019) Physical Activity Questionnaire for Older Children (PAQ-C) with 10 questions adapted into Turkish was used. Additionally, the Validity and Reliability of the Game Addiction Scale for Adolescents-Short Form game addiction scale developed by Gazanfer and Taş (2018) was used to determine game addiction levels.

### Body mass index

Anthropometric measurements were measured standing height and body weight with light clothing and no shoes. In this direction, BMI values were calculated by measuring the height and weight of the individuals; The 85th and 95th percentiles were considered overweight, and those above the 95th percentile were considered obese (Neyzi et al., 2008; Ogden and Flegal, 2010; Wei et al., 2020; Cdc "Centers for Disease Control and Prevention (CDC), 2020, 2022) determining the BMI values and obesity status of the participants, the child body mass index calculation application on the website of Centers for Disease Control and Prevention (CDC) was used (CDC). The outcomes extracted for further data treatment was the BMI measured in kg/m^2^.

### Digital game addiction questionnaire

The scale developed by Lemmens et al. (2009) was adapted into Turkish by Gazanfer and Taş (2018). Scale consists of nine items. 5-point Likert-type grading was used to score the scale. The lowest score to be obtained from the scale is 9 and the highest score is 45. The grading is “Never,” “Rarely,” “Sometimes,” “Often,” and “Very often.” The Cronbach Alpha internal consistency coefficient of the scale was found to be 0.92. These results show that the responses to our scale were consistent before further to statistical analysis. The total scale score was used in the evaluation of digital game addiction.

### Physical activity questionnaire for older children

The scale consists of 10 items, of which nine items are used to determine the level of physical activity. The tenth item gives information about whether the participant does physical activity in case of illness. The tenth item is excluded from the calculation of the physical activity score. The first question in PAQ C provides descriptive options for what they do physically in 22 titles. Responses to this question are evaluated as a 5-point rating (1 = no activity, 5 = 7 times or more), from which the average score is calculated; higher scores indicate greater levels of physical activity. Defining the 22 activities clearly and precisely will be a reminder to the children. The other eight questions relate to the assessment of physical activities performed during the day or at specific time intervals throughout the week (e.g., physical education class, recess, noon, after-school, evening, weekend). These items are scored on a 5-point scale, with higher scores indicating higher activity level. The overall PAQ-C score is obtained by adding the scores of items 1–9, and the final physical activity-level score is the average of the scores of these nine items. An average of 1 point indicates a low PA level, and an average of 5 points indicates a high PA level (Kowalski et al., 2004; Erdim et al., 2019). The total scale score was used in the evaluation of physical activity level.

### Statistical analysis

The conformity of the variables to the normal distribution was examined using visual (histogram and probability plots) and analytical (Shapiro–Wilk Test) methods. The assumption of homogeneity of variances was examined by Levene’s test. Descriptive statistics are expressed as median, interquartile range for non-normally distributed variables, and mean ± standard deviation for normally distributed variables. Independent Samples t-test was used in the comparisons of two groups regarding the variables satisfying the parametric test assumptions. Mann–Whitney *U* and Kruskal–Wallis H tests were used for comparisons of two or more groups regarding the variables that did not meet the parametric test assumptions, respectively. Frequency (*n*) and percentage (%) values were calculated for qualitative variables. Comparison of BMI levels with physical activity and game addiction scale scores was done using the Kruskal–Wallis H analysis. After the Kruskal–Wallis H test results, the Conover test was used for pairwise comparisons for universally significant variables. Effect size was calculated using the Cohen’s *D*. The magnitude of effect size was considered following the thresholds: Cohen suggested that *d* = 0.2 be considered a ‘small’ effect size, 0.5 represents a “medium” effect size and 0.8 a “large” effect size (Cohen, 1988). Relationships between outcomes were conducted using the Pearson-r product moment correlation test. The magnitude of correlations were defined as follows: <0.1 = trivial, 0.1–0.3 = small, 0.3–0.5 = moderate, 0.5–0.7 = large, 0.7–0.9 = very large, and >0.9 = nearly perfect (Batterham and Hopkins, 2006). In multivariate analysis, independent predictors were analyzed using binomial logistic regression analysis and possible factors identified in previous analyses. Logistic regression was performed according to the forward feature selection method. Hosmer-Lemeshow and Omnibus tests were used to evaluate the logistic regression model and its coefficients. In all results, *p*-value of <0.05 was considered statistically significant. American Psychological Association (APA) 6.0 style was used to report statistical differences (Yağin et al., 2021). Statistical analyzes were performed using Python 3.9 software and SPSS 28.0 (IBM Corp., Armonk, NY, United States) package program.

### Data preprocessing and machine learning approach

This section provides a description of the approach used to evaluate the predictive ability of the machine learning (ML) approach for game addiction prediction. In the study, there was a high level of class imbalance problem in the distribution of the groups for digital game addiction [digital game addiction: 38 (9.4%), no-digital game addiction 367 (90.6%)]. Synthetic Minority Over-sampling Technique for Nominal and Continuous (SMOTE-NC) Gök and Olgun (2021) was used to eliminate the class imbalance problem. Class imbalance problem, when working with real-life data, this problem is highly prevalent and can be defined as a state of imbalance when there are significantly more cases belonging to the majority class than those belonging to the minority class (Paksoy and Yağin, 2022). Because ML techniques like logistic regression might be biased toward the majority class, which causes issues with under or overfitting, balanced data is crucial. The standard oversampling method multiplies the available data, but the SMOTE makes synthetic samples from the minority class using the information in the data, and under sampling eliminates the majority class from the data. As a result, SMOTE is a popular technique for imbalance concerns since it may perform better than straightforward sampling techniques by avoiding over or underfitting difficulties (Chen et al., 2022). Afterwards, a logistic regression model was created based on the advanced feature selection method, and accuracy, sensitivity, specificity, FI-score, positive and negative predictive values were calculated using the Python 3.9 program with the help of the confusion matrix to evaluate the prediction performance of the model.

## Results

The mean age of the participants was 11.38 ± 1.45 years. Of the 405 participants, 231 (57%) were girls and 174 (43%) were boys. The mean age was 11.28 ± 1.47 years for girls participants and 11.51 ± 1.43 years for boys participants. The mean BMI of the participants was 19.48 ± 4.13, and 37 (9.14%) severely underweight, 45 (11.11%) underweight, 187 (46.17%) healthy weight, 76 (18.77%) overweight, and 60 (14.81%) was in the obesity category. The mean total physical activity score of the participants was 2.96 ± 0.82, the mean total out of school score was 2.78 ± 0.94, the mean total school-based score was 3.31 ± 1.05, and the mean digital game addiction score was 1.89 ± 0.70 (Table 1).

**Table 1:** Descriptive statistics.

Variable	Statistics	Value
Gender		
Girls		231.00 (57.04)
Boys		174.00 (42.96)
BMI		
Severely underweight	*n* (%)	37.00 (9.14)
Underweight		45.00 (11.11)
Healthy weight		187.00 (46.17)
Overweight		76.00 (18.77)
Obesity		60.00 (14.81)
Age (year)	M ± SD	11.37 ± 1.45
Height (cm)		149.42 ± 11.16
Weight (kg)		44.22 ± 13.06
TPA		2.96 ± 0.82
TPA-OS		2.78 ± 0.94
TPA-SB		3.31 ± 1.05
DGA		1.89 ± 0.70

BMI, body mass index; TPA, total physical activity; TPA-OS, total physical activity out of school; TPA-SB, total physical activity-school based; DGA, digital game addiction; M, mean; SD, standard deviation.

According to the findings of the study, age, height, and TBA scores of men and women were similar (*p* > 0.05). Body mass, TBA-OS, and TBA-SB scores, and DGA scores of women were significantly higher than men (*p* < 0.05). In addition, gender was significant for BMI and was higher in boys (*p* < 0.05; Table 2).

**Table 2:** The variation of anthropometric variables, physical activity, and digital game addiction scores by gender.

Variables	Boys (*n* = 174)	Girls (*n* = 231)	*p*-Value	ES
Anthropometric				
Age (year)*	12 (3)	12 (3)	0.16	0.14
Height (cm)**	148.83 ± 10.77	150.21 ± 11.65	0.22	0.12
Body mass (kg)*	41 (18)	45 (21)	**0.01**	0.24
BMI (kg/m^2^)**	20.21 ± 4.56	18.93 ± 3.67	**0.002**	0.31
**Physical activity**				
TBA**	3.00 ± 0.85	2.90 ± 0.78	0.26	0.11
TPA-OS*	2.66 (1.50)	2.87 (1.16)	**0.03**	0.21
TPA-SB*	3.00 (1.66)	3.33 (1.33)	**0.04**	0.20
**Game**				
DGA*	1.66 (1.00)	1.94 (0.78)	**0.003**	0.29

Significant results are in bold. BMI, body mass index; TPA, total physical activity; TPA-OS, total physical activity out of school; TPA-SB, total physical activity-school based; DGA, digital game addiction; ES, effect size.

* Mann–Whitney *U* test (variables summarized by median and interquartile range).

** Independent samples *t*-test (variables summarized with mean and standard deviation).

Participants’ age, body mass, and BMI were significantly higher in the DGA group (*p* < 0.05). TPA and TPA-OS scores were similar in the groups (*p* > 0.05), but the TPA-SB score was higher in the non-DGA group (*p* < 0.05; Table 3).

**Table 3:** Results of anthropometric variables, physical activity and digital game addiction scores according to game addiction.

Variables*	No-DGA (*n* = 367)	DGA (*n* = 38)	*p*-Value	ES
Anthropometric				
Age (year)	11 (3)	12 (2)	**0.019**	0.23
Height (cm)	150 (17)	153.50 (15.00)	0.061	0.19
Body mass (kg)	42 (19)	52.50 (20.00)	**0.001**	0.38
BMI (kg/m^2^)	18.77 (5.86)	22.10 (6.01)	**0.001**	0.34
**Physical activity**				
TPA	3.00 (1.11)	2.66 (0.77)	0.077	0.18
TPA-OS	2.66 (1.33)	2.50 (1.16)	0.238	0.12
TPA-SB	3.33 (1.33)	3.00 (1.33)	**0.049**	0.19

Significant results are in bold. BMI, body mass index; TPA, total physical activity; TPA-OS, total physical activity out of school; TPA-SB, total physical activity-school based; DGA, digital game addiction; ES, effect size.

* Mann–Whitney *U* test (Variables summarized by median and interquartile range).

Table 4 shows the changes in physical activity and game addiction scale scores in BMI groups. Results showed that TPA, TPA-OS and TPA-SB scores were similar in BMI groups (*p* > 0.05). However, there was a statistically significant difference between the BMI groups in terms of DGA score, and further analysis revealed that the DGA score was significantly higher in the obesity group compared to the healthy weight group (*p* = 0.025; ES: 0.27).

**Table 4:** Changes in BMI groups for physical activity and DGA scores.

Variable**	BMI Group*					*p*-Value^#^	ES
	Severely underweight (*n* = 37)	Underweight (*n* = 45)	Healthy Weight (*n* = 187)	Overweight (*n* = 76)	Obesity (*n* = 60)		
**Physical activity**							
TPA	3.11^a^ (1.22)	2.66^a^ (1.11)	2.88^a^ (1.16)	3.00^a^ (1.03)	3.11^a^ (1.16)	0.19	0.14
TPA-OS	3.00^a^ (1.33)	2.50^a^ (1.33)	2.66^a^ (1.41)	2.66^a^ (1.50)	2.83^a^ (1.04)	0.23	0.12
TPA-SB	3.33^a^ (1.00)	3^a^ (1)	3.00^a^ (1.33)	3.00^a^ (1.66)	3.66^a^ (1.41)	0.25	0.11
**Game addiction**							
DGA	1.77^ab^ (0.88)	1.88^a^ (0.66)	1.66^b^ (0.83)	1.88^ab^ (0.88)	1.88^a^ (1.00)	**0.025**	0.27

Significant results are in bold. TPA, total physical activity; TPA-OS, total physical activity out of school; TPA-SB, total physical activity-school based; DGA, digital game addiction.

* There is a statistically significant difference in group categories that do not contain the same letter in each row (Conover test; *p* < 0.05).

** Variables are summarized as “median (interquartile range).”

# Kruskal Wallis *H* test.

The results of the logistic regression model established with the forward feature selection method are given in Table 5. Age, gender, BMI and TPA scores, the coefficients of which were statistically significant in the model, were determined to be predictors of game addiction. The resulting logistic regression equation is as follows:


$$Logit\;\left(P\right)=1/\left(1+\exp\left(-\left(\begin{array}{l} -3.384+{\mathrm{Age}}^{*}\;0.124\\ +\mathrm{Gender}-{\mathrm{boys}}^{*}\;\left(-0.953\right)\\ +{\mathrm{BMI}}^{*}\;0.145+{\mathrm{TPA}}^{*}\;\left(-0.410\right) \end{array}\right)\right)\right)$$

**Table 5:** Results of the logistic regression model created to predict DGA.

Variable	B	SE	Wald	*p*-Value	OR	95% CI for OR		Interpretation
						Lower	Upper	
Age (year)	0.124	0.061	4.126	0.042	1.132	1.004	1.277	Increasing effect
Gender-boys	−0.953	0.174	30.062	<0.001	0.386	0.274	0.542	Reducing effect
BMI (kg/m^2^)	0.145	0.023	39.327	<0.001	1.157	1.105	1.210	Increasing effect
TPA	−0.410	0.116	12.402	<0.001	0.664	0.528	0.834	Reducing effect
Constant	−3.384	0.824	16.886	<0.001	0.034	–	–	

BMI, body mass index; TPA, total physical activity; B, coefficient; SE, standard error; OR, odds ratio; CI, confidence interval.

Our results revealed that one unit increase in age increased DGA risk by 1.132 times (OR = 1.132; 95% CI [1.004–1.277]; *p* = 0.042). Moreover, women had a 2.59-fold greater risk of DGA compared to men (OR = 1.132; 95% CI [1.004–1.277]; *p* = 0.042). BMI was a predictor of DGA, and a one-unit increase in BMI increased DGA risk 1.105-fold (OR = 1.132; 95% CI [1.004–1.277]; *p* = 0.042). Furthermore, physical activity was found to be an important factor reducing DGA 1.51-fold (OR = 0.664; 95% CI [0.528–0.834]; *p* = 0.035; Table 5).

In Table 6, the results of the performance criteria for the prediction of game addiction of the logistic regression model are reported. The accuracy rate calculated in the DGA estimation is 75.3% and it can be said that the prediction performance of the model is quite high.

**Table 6:** Performance criteria for the DGA predict model.

Metrics	Value (95% CI)
Accuracy	0.753 (0.722–0.785)
Sensitivity	0.747 (0.700–0.790)
Specificity	0.760 (0.712–0.803)
Positive predictive value	0.766 (0.719–0.808)
Negative predictive value	0.741 (0.693–0.785)
F1-score	0.756 (0.725–0.787)

CI, confidence interval.

**Figure 1:**
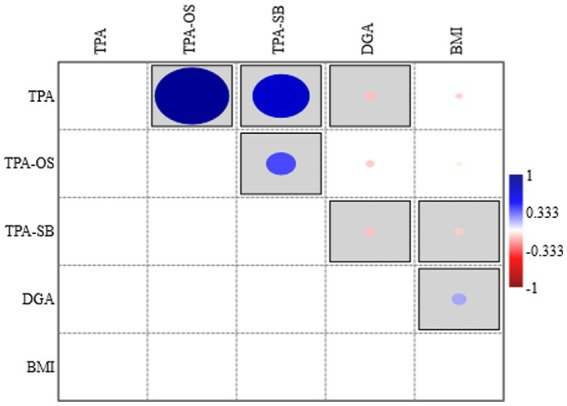
The correlation graph between TPA, TPA-OS, TPA-SB, DGA scores, and BMI. TPA, total physical activity; TPA-OS, total physical activity out of school; TPA-SB, total physical activity-school based; DGA, digital game addiction; the correlation coefficients shown in the gray box represent statistical significance (*p* < 0.05); the size of the circle in the gray box indicates the level of correlation; blue color indicates positive and red color negative correlation.

**Figure 2:**
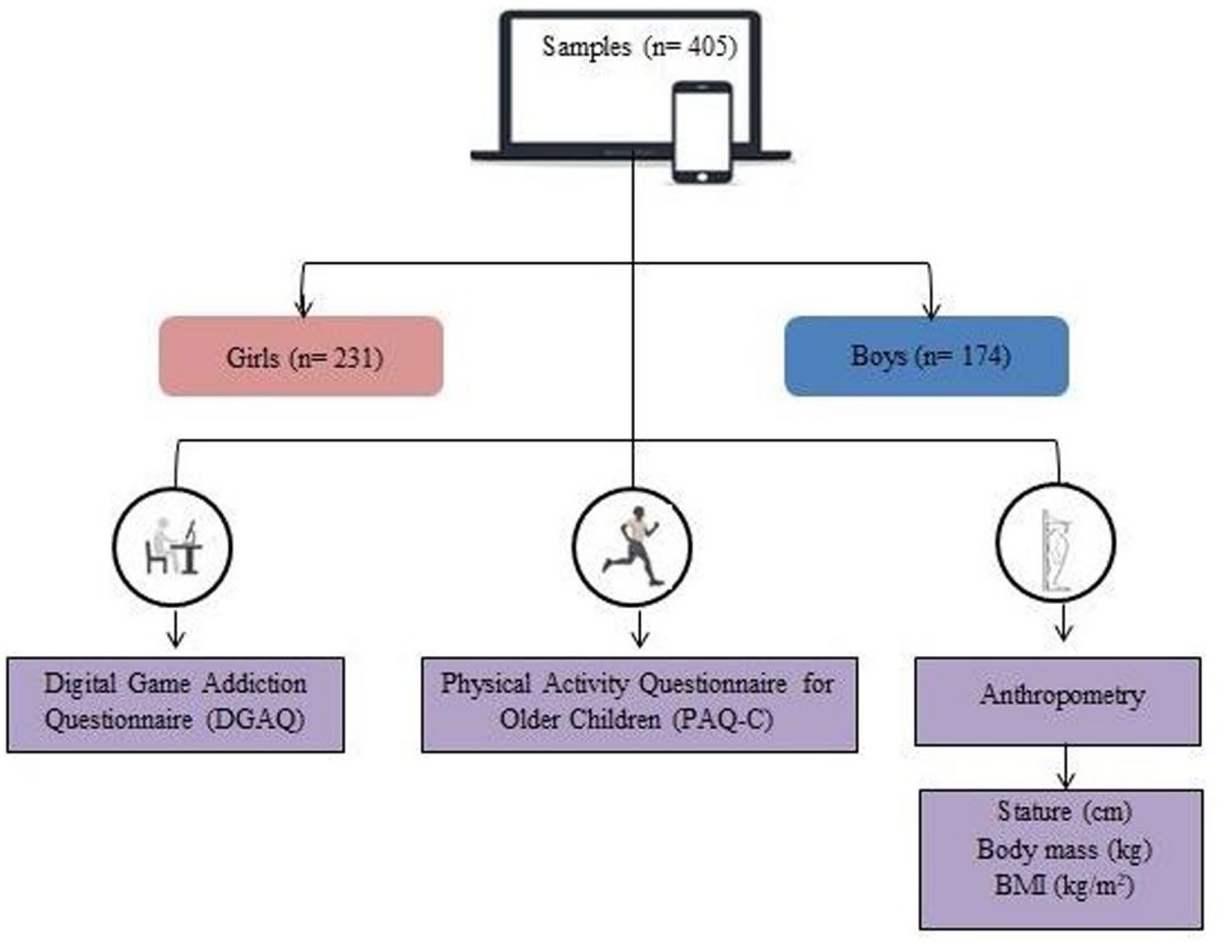
Study design.

Figure 1 shows the results of the correlation analysis. When Figure 2 was examined, a small negative correlation was found between DGA and TPA, TPA-SB scores. As a results, as TPA and TPA-SB scores increase, DGA scores decrease (*p* < 0.05). There was a small negative correlation between BMI and TPA-SB, while a small positive correlation was observed between BMI and DGA scores (*p* < 0.05).

## Discussion

The primary aim of this study was to define the prevalence of obesity, physical activity levels and digital game addiction level in adolescents, and to investigate gender differences and relationships between outcomes. The second aim was to predict game addiction based on anthropometric measurements and physical activity levels. According to our findings, it was determined that digital game addiction has a negative relationship with physical activity level. We found that physical activity level had a negative relationship with BMI. In addition, physical activity reduces obesity and DGA. Game addiction levels of girl participants were significantly higher than boy participants, and we also found that obesity increased DGA. With the data obtained, it was determined that age, gender, BMI and total physical activity (TPA) score predicted DGA. The results showed that as age and BMI increase the risk of DGA, girls were 2.59-fold more likely than boys to develop DGA. More importantly, the results of this study showed that physical activity was an important factor in reducing DGA 1.51-fold. Our prediction model: Logit (P) = 1/(1 + exp(−(−3.384 + Age * 0.124 + Gender-boys * (−0.953) + BMI * 0.145 + TPA * (−0.410)))).

In a study, the game addiction levels of girls were found to be higher than boys. This result supports our study, and in our findings, it was determined that the game addiction levels of girl participants were higher than boy participants. However, in another study, the problematic internet use behavior of boys were found to be higher than girls (Durmus et al., 2021). According to studies, problematic internet behaviors are more common in adolescent boys than in adults and adolescent girls (Hancox et al., 2004; Tsai et al., 2009). This may be due to the negative effects of adolescence period. The reasons for the similarities and differences in the literature and our findings may be different due to factors such as the period in which the research was conducted, the welfare level of the country in which the research was conducted, and the region (rural or urban) in which the research was conducted. In addition, the main reason for the conflicting results in the literature may be due to factors such as the period of the research (e.g., pandemic). In another study on smartphone use, the daily calorie consumption, the number of steps taken per day were evaluated for individuals more screen time, and it was reported that they were more sedentary than those who spent less time in front of the screen (Kim et al., 2015). This result shows results in line with our findings. In this study, it was an expected result that there was a decrease in the level of physical activity with the increase in game addiction. In a study that supports our findings, physical activity is an important factor in solving the problem of digital game addiction (Hazar et al., 2017). Another study shows that there is a positive and significant relationship between middle school students’ physical activity participation and awareness of digital game addiction (Çar and Ahraz, 2022). It is possible that a study may have found that physical activity is an important factor in solving the problem of digital game addiction. In this study and literature may have found a positive and significant relationship between physical activity participation and awareness of digital game addiction. It is possible that engaging in physical activity may help individuals who are struggling with digital game addiction by providing a healthy outlet for stress and anxiety, and by promoting self-regulation and self-control. It is also important to note that, as with any study, it is important to consider the limitations and the sample size, and how generalizable the findings are to other populations before making a definitive conclusion. Additionally, it is important to keep in mind that digital game addiction is a complex issue and different interventions will be needed for different individuals, including psychological and social support, as well as education and awareness. Studies have shown that problematic internet use is associated with a high BMI and a sedentary lifestyle (Kautiainen et al., 2005; Tao, 2013). In parallel, another study reported associations between excessive internet, cell phone use, and long screen time and obesity among Saudi school-aged children (Alturki et al., 2020). These results are similar to our study. However, in another study in contrast, no correlation was found between problematic internet use and BMI and PA values in both girls and boys (Durmus et al., 2021). This could be due to a variety of factors such as differences in study design, sample size, and population characteristics. In the study Durmus et al. (2021) the researchers did not find a correlation between problematic internet use and BMI and PA values in both girls and boys. This suggests that other factors, such as diet and genetics, may play a larger role in the development of obesity among individuals who engage in problematic internet use.

Another study reported that a relationship between problematic internet use behavior and age variables in adolescents (Doğan, 2013; Al-Gamal et al., 2016). In another study, problematic internet use behavior was found to be lower in the 10–14 age group than in the 15–19 age group (Durmus et al., 2021). The different results in the literature are probably due to other factors (e.g., obesity can also be caused by nutritional status and some health problems). In addition, physical activity may differ depending on the region of residence. As a matter of fact, a study found that physical activity differs between rural and urban children (Gülü et al., 2022).

In addition to physical activity, diet has a positive effect on preventing obesity (Hills and Byrne, 2006). Norshakirah et al., found that Digital addiction among Malaysian adolescents can cause various impacts on physical health such as obesity, physical inactivity. These results in line with our findings. In this study from the data obtained, it was determined that age, gender, BMI and total physical activity (TPA) score predicted game addiction. The similarity between the findings of this study and those in the literature suggests that a sedentary lifestyle is probably one of the factors that cause obesity. It may also be that game addiction triggers a sedentary life. Another study found that obese children had lower physical activity levels than children with normal BMI (Gülü et al., 2022). Along with regular physical activity during the developmental period of the child, access to healthy food and proper nutrition is probably important determinants in the development of the physical structure of each child, in line with their genetic potential (Hills et al., 2007; Sallis and Glanz, 2009). In a study, it was found that the prevalence of obesity in adolescents was quite high (37.6% in boys, 32.9% in girls; Durmus et al., 2021). These results are also consistent with our findings.

When we examine it as participation in physical activity, in the literature, adolescents exhibit a sedentary lifestyle, there is a significant relationship between physical activity rates and gender, and boys are more energetic than girls (Feldman et al., 2003; Yılmaz et al., 2014; Durmus et al., 2021). There appears to be a strong association between physical activity and obesity in children and adolescents (Hills et al., 2011). This result directly support our research. In this study, negative relationship was found between BMI and physical activity level. The effect of physical activity on BMI was limited in this study, meaning that it alone may be insufficient to prevent obesity. For this reason, to prevent obesity in children, it is necessary to increase participation in physical activity and to control their nutritional status. Future studies to determine the underlying causes of obesity may conduct more detailed studies on the nutritional status of children and physical activity level.

A limitation of this study is its cross-sectional methodology. Unfortunately, we were unable to collect longitudinal data in this first part of the study. Future research should include a longitudinal method to better represent the developmental course of this proposed disorder. Another our limitation is that the generalizability of the findings may be limited as the participants of this study were limited to Turkish students and the sample size was small. Additionally, since this study was conducted only on adolescent children, not all results can be generalized to the population. Finally, the findings of this study may be reporting bias due to the use of a self-report scale in data collection. More effective results could have been obtained if we had the opportunity to use smart wristbands that measure activity by providing more precise data, especially in the evaluation of physical activity. On the other hand, another limitation of this study is that obesity status was performed using height and body weight according to the WHO classification, and if we had the possibility of screening with a gold standard method in determining obesity in this study, it would have strengthened our results. In future studies, the effect of digital game addiction can be investigated in more detail by methods that can analyze self-efficacy, mental, and physical health conditions in more detail.

## Conclusion

The main finding of this study is that as the level of physical activity increases, game addiction will decrease. Regular physical activity should be encouraged and digital gaming hours can be limited to maintain ideal weight. In addition, adolescents should be encouraged to engage in physical activity in order to reduce digital game addiction level. This research provides important information in the prevention of DGA and obesity by increasing physical activity. According to these outputs, policy makers should carry out projects to increase participation in physical activity in schools and outside of school in order to reduce the time spent by adolescents on the screen in today’s technology age.

## Data availability statement

The raw data supporting the conclusions of this article will be made available by the authors, without undue reservation.

## Ethics statement

The studies involving human participants were reviewed and approved by Kirikkale University Social and Human Sciences Ethics Committee. Written informed consent to participate in this study was provided by the participants’ legal guardian/next of kin.

## Author contributions

MG: conceptualization and methodology. IG: data collection. EA, HY, and PP-G: analysis. MG, FY, and HN: writing—original draft preparation. MG, FC, AZ, LPA, PP-G, and HN: writing—review and editing. All authors have read and agreed to the published version of the manuscript.

## Funding

FC and this work are funded by the Fundação para a Ciência e Tecnologia/Ministério da Ciência, Tecnologia e Ensino Superior through national funds, and when applicable, co-funded by EU funds under the project UIDB/50008/2020.

## Conflict of interest

The authors declare that the research was conducted in the absence of any commercial or financial relationships that could be construed as a potential conflict of interest.

## Publisher’s note

All claims expressed in this article are solely those of the authors and do not necessarily represent those of their affiliated organizations, or those of the publisher, the editors and the reviewers. Any product that may be evaluated in this article, or claim that may be made by its manufacturer, is not guaranteed or endorsed by the publisher.

## Abbreviations

COVID-19: Coronavirus disease
BMI: Body mass index
WHO: World Health Organization
PAQ-C: Physical Activity Questionnaire for Older Children
YFAS-C: Yale Food Addiction Scale for Children
CDC: Disease control and prevention
DGA: Digital game addiction
